# Dendritic cell-derived exosomes (Dex) are potential biomarkers of response to Ipilimumab in metastatic melanoma

**DOI:** 10.1186/1479-5876-13-S1-P15

**Published:** 2015-01-15

**Authors:** Stefania Stucci, Marco Tucci, Paolo Antonio Ascierto, Anna Passarelli, Capone Mariaelena, Gabriele Madonna, Simeone Ester, Antonio Maria Grimaldi, Franco Silvestris

**Affiliations:** 1 Medical Oncology Unit - Department of Biomedical Sciences and Clinical Oncology, University of Bari, Italy; 2 Melanoma, Cancer Immunotherapy and Innovative Therpay Unit - INT “G.Pascale”, Napoli, Italy

## Background

Chemotherapy and vaccination with tumor-loaded dendritic cells (DCs) show poor impact on overall survival (OS) in metastatic melanoma^1,2^. Ipilimumab (IPI) improves OS throughout the blockade of CTLA4-mediated inhibitory signals in T-cells and restores the efficiency of the antigenic cross-priming by mature (m) DCs^3^. However, variation of immune cells to evaluate the response to IPI does not reflect the T-cell activation and is a modest predictor of clinical response. Recent studies in human and experimental melanoma demonstrated that mDCs release endomysial microvescicles namely dexosomes (Dex) showing a functional anti-melanoma activity as well as an antigenic profile resembling that of circulating mDCs including CD40, CD80 and CD86 co-stimulatory molecules.

This research is aimed to identify an early biomarker of T-cell activation for predicting the clinical response in IPI-treated melanoma patients.

## Methods

Thirthy-four patients with metastatic melanoma were treated with IPI and sera collected at each infusion. Serum Dex were purified by the ‘Total Exosome Isolation kit’ (Invitrogen) and conjugated with magnetic beads of 4 μm of diameter (Dynabeads). Dex were first identified by size and then CD40, CD80 and CD86 expression was evaluated by flow-cytometry using relative MoAbs. The response to IPI was analyzed up to 12 weeks after the end of treatment, according to RECIST criteria. Moreover, DEX levels were compared with clinical and immunological parameters by the Mann-Withnet test.

## Results

Both CD40 and CD80 expression was unchanged after the end of IPI treatment with respect to basal levels, whereas a significant increase of Dex-CD86 expression occurred as compared to baseline (21.3±1.5% vs 12.0±0.8%, p<0.05) in 5 patients with partial response and 1 in complete remission (19.4±1.2% vs. 9.9±1.1%). A weak trend to the increment of Dex-CD86 occurred in patients with stable disease, while those in clinical progression showed low levels in all instances. CD86 expression was apparently unrelated to LDH levels and absolute leukocyte count.

## Conclusions

Level of CD86 expressed by Dex reflects the immunological activation in melanoma patients treated with IPI. Therefore, the measurement of soluble Dex-CD86 could be an early marker of response to IPI and predict the efficiency of immunological response.

